# Supplementary material for: Experiences of a web-based psycho-educational intervention targeting sexual dysfunction and fertility distress in young adults with cancer—A self-determination theory perspective
Source: PLoS One. 2020 Jul 22;15(7):e0236180. doi: 10.1371/journal.pone.0236180 (PMC7375532; doi:10.1371/journal.pone.0236180)
Supplement: S1 File — (DOCX) [file pone.0236180.s001.docx]

| No. | Item | Description | Answer | Appears in Section# |
| --- | --- | --- | --- | --- |
| **Domain 1: Research team and reflexivity** | | | | |
| Personal characteristics | | | | |
| 1. | Interviewer/facilitator | Which author(s) conducted the interview or focus group? | All authors | Methods |
| 2. | Credentials | What were the researchers’ credentials? *E.g. PhD, MD* | CMO=PhD Student; LL, CL, LW, LEE=PhD |  |
| 3. | Occupation | What was their occupation at the time of the study? | CMO=PhD Student; LL=postdoc; CL, LW, LEE=senior lecturers and associate professors |  |
| 4. | Gender | Were the researchers male or female? | CMO, LL, CL, LW=female LEE=male |  |
| 5. | Experience and training | What experience or training did the researchers have? | Training in interview techniques and qualitative content analysis. Some had extensive experience of interviewing and qualitative content analysis. | Methods |
| Relationship with participants | | | | |
| 6. | Relationship established | Was a relationship established prior to study commencement? | No, with the exception of one interviewee who had had a previous personalized telephone consultation with the interviewer, as part of the program. |  |
| 7. | Participant knowledge of the interviewer | What did the participants know about the researcher?  *E.g. Personal goals, reasons for doing the research* | That the researchers had conceived and participated in the management of the RCT. | Methods |
| 8. | Interviewer characteristics | What characteristics were reported about the interviewer/facilitator?  *E*.g*. bias, assumptions,* *reasons and interests in the research topic* | Interviewers had been part of the research team both conceiving and evaluating the intervention. | Methods |
| **Domain 2: Study design** | | | | |
| Theoretical framework | | | | |
| 9. | Methodological orientation and theory | What methodological orientation was stated to underpin the study? *E.g. grounded theory, discourse analysis, ethnography, phenomenology, content analysis?* | Framework approach for thematic analysis, abductive approach | Methods |
| Participant selection | | | | |
| 10. | Sampling | How were participants selected? *E.g. purposive*, *convenience,* *consecutive, snowball?* | Purposive sampling | Methods |
| 11. | Method of approach | How were participants approached? *E.g. face-to-face, telephone, mail, email* | Email followed by telephone call | Methods |
| 12. | Sample size | How many participants were in the study? | 28 | Methods |
| 13. | Non-participation | How many people refused to participate and how many dropped out? What were the reasons for this? | 29 people could not be reached or actively refused. Three dropped out before being interviewed. Reasons for refusal (n=29) or dropout (n=3) were not investigated unless the person spontaneously brought it up. Stated reasons were lack of time, not having been an active user, or lack of interest. | Methods |
| Setting | | | | |
| 14. | Setting of data collection | Where was the data collected? *E.g. home, clinic, workplace* | The participants took part in the telephone interview at a location of the participants’ choice, typically in the participants’ home. | Methods |
| 15. | Presence of non-participants | Was anyone else present besides the participants and researchers | Possibly in the case of participants. Since the interviews were conducted over the telephone, this was out of the researchers’ control. However, the participants were encouraged to make sure they would not be disturbed during the interview. | Methods |
| 16. | Description of sample | What are the important characteristics of the sample? *E.g. demographic data, date* | Described in table 1. | Methods |
| Data collection | | | | |
| 17. | Interview guide | Were questions, prompts, guides provided by the authors? Was it pilot tested? | Yes. The interview guide, both the original Swedish version and translated into English, is enclosed as a supplementary file. The final interview guide was not pilot tested on the target population but had been tested in an educational setting (PhD course in interview techniques) before conducting the data collection. | Methods, Supplementary file |
| - | Repeat interviews | Were repeat interviews carried out? If yes, how many? | No | - |
| 19. | Audio/visual recording | Did the researchers use audio or visual recording to collect the data? | Yes, audio recording was used | Methods |
| 20. | Field notes | Were field notes made during and/or after the interviews or focus group? | Yes | Methods |
| 21. | Duration | What was the duration of the interviews or focus group? | 20-60 minutes | Methods |
| 22. | Data saturation | Was data saturation discussed? | No, N.A. according to the methodology (thematic analysis) |  |
| 23. | Transcripts returned | Were transcripts returned to participants for comment and/or correction | No. However, participant validation was obtained at the end of each interview by summarizing the experiences each participant had shared and asking if they had anything to add or clarify. | Methods |
| **Domain 3: Analysis and findings** | | | | |
| Data analysis | | | | |
| 24. | Number of data coders | How many data coders coded the data | Two researchers (first and last author) did the data reduction/coding but the ensuing framework matrices and their interpretation were repeatedly discussed by the whole research team. | Methods |
| 25. | Description of the coding tree | Did authors provide a description of the coding tree? | Yes/N.A. Framework methodology was used, its various steps of data reduction are described. | Methods |
| 26. | Derivation of themes | Were themes identified in advance or derived from the data? | Both, since we applied an abductive approach. Main themes were deductively applied according to the theoretical orientation of the intervention | Methods, Discussion |
| 27. | Software | What software, if applicable, was used to manage the data? | NVivo 12 | Methods |
| 28. | Participant checking | Did participants provide feedback on the findings? | No |  |
| Reporting | | | | |
| 29. | Quotations presented | Were participant quotations presented to illustrate themes/findings? Was each quotation identified? *E.g. participant number* | Yes | Results |
| 30. | Data and findings consistent | Was there consistency between the data presented and the findings? | Yes | Results, Discussion |
| 31. | Clarity of major themes | Were major themes clearly presented in the findings? | Yes | Results, Discussion |
| 32. | Clarity of minor themes | Is there a description of diverse cases or discussion of minor themes? | Yes | Results, Discussion |

Developed from: Tong A, Sainsbury P, Craig J. Consolidated criteria for reporting qualitative research (COREQ): a 32-item checklist for interviews and focus groups. International Journal for Quality in Health Care. 2007. Volume 19, Number 6: pp. 349 – 357
