# Supplementary material for: Experiences of a web-based psycho-educational intervention targeting sexual dysfunction and fertility distress in young adults with cancer—A self-determination theory perspective
Source: PLoS One. 2020 Jul 22;15(7):e0236180. doi: 10.1371/journal.pone.0236180 (PMC7375532; doi:10.1371/journal.pone.0236180)
Supplement: S2 File — (DOCX) [file pone.0236180.s002.docx]

**Supplementary file 1**

**Selected interview questions**

*Could you tell me about your current treatment status?*

*Are you working or studying now?*

*What is your family situation like?*

*What has been the toughest or most distressing part for you concerning sexuality/fertility after cancer?*

*How do these thoughts affect you?*

*Do you think anything has changed in the past couple of months? In what way?*

*If things are more or less like before, why do you think that is? (The lack of change).*

*Did you seek any kind of help or support before starting the program?*

*To what extent would you say you have been using the program? (a lot, a little or not at all?)*

*In what ways did you use the program? (Relevant probes)*

*How come you didn’t use the program?*

*How did the program affect you?*

*How do you think it should have been designed in order to specifically appeal more to you?*

*The program deliberately provides quite a lot of information. How did you feel about that?*

*Do you think you learnt anything from the program? (Tell me more!)*

*What did you think about the exercises in the program?*

*What did you think about hearing other people’s stories?*

*Did you receive any concrete advice that you were able to use in your personal situation? What did you do?*

*Was there anything about the program that made you feel uncomfortable?*

*Would you have wished something more or something different from the program?*

*The program is partly about relationships. What do you think it would have been like if both you and a partner had had access to the program?*

*Did any other important events take place in your life these past couple of months?*

*Is there anything you would like to add that you think I should have addressed?*

**Interview questions in Swedish**

*Vill du berätta var du just nu står när det gäller behandlingar?*

*Är du igång med arbete eller studier nu?*

*Hur ser din familjesituation ut?*

*Vad har varit jobbigast/har du varit mest orolig för när det gäller sexualitet/fertilitet efter cancer?*

*Hur påverkar de här tankarna dig?*

*Tycker du att något har förändrats de senaste månaderna? På vilket sätt?*

*Om det är ungefär som innan, vad tänker du att det beror på? (att inget har förändrats).*

*Hade du sökt någon hjälp eller stöd innan du började programmet?*

*Hur mycket skulle du säga att du har använt programmet? (mycket, lite eller inget alls)*

*Hur har du gjort när du har använt programmet? (Relevanta följdfrågor)*

*Hur kom det sig att du inte använde programmet?*

*Hur har programmet påverkat dig?*

*Hur skulle programmet ha utformats så att det lockat just dig att använda det mer?*

*Programmet är uppbyggt kring att man får ganska mycket information. Hur var det för dig?*

*Tycker du att du har lärt dig något nytt av programmet? (Berätta mer!)*

*Vad tyckte du om övningarna i programmet?*

*Hur har du tyckt det har varit att ta del av andras berättelser?*

*Fick du i programmet några konkreta råd eller tips som du har kunnat använda i just din situation? Vad gjorde du då?*

*Har något i programmet varit jobbigt eller obehagligt?*

*Hade du önskat något mer eller något annat av programmet?*

*Programmet handlar ju till en del om relationer. Hur skulle det varit om både du och en partner hade haft tillgång till programmet tror du?*

*Har något annat viktigt hänt i ditt liv under de här månaderna?*

*Är det något du vill lägga till som du tycker att jag borde ha tagit upp?*
